# A Comprehensive Review on Studying and Developing Guidelines to Standardize the Inspection of Properties and Production Methods for Mycelium-Bound Composites in Bio-Based Building Material Applications

**DOI:** 10.3390/biomimetics9090549

**Published:** 2024-09-11

**Authors:** Worawoot Aiduang, Praween Jinanukul, Wandee Thamjaree, Tanongkiat Kiatsiriroat, Tanut Waroonkun, Saisamorn Lumyong

**Affiliations:** 1 Office of Research Administration, Chiang Mai University, Chiang Mai 50200, Thailand; worawoot.aiduang@cmu.ac.th; 2 Department of Biology, Faculty of Science, Chiang Mai University, Chiang Mai 50200, Thailand; 3 Faculty of Architecture, Chiang Mai University, Chiang Mai 50200, Thailand; praween_ji@cmu.ac.th; 4 Department of Physics and Materials Science, Faculty of Science, Chiang Mai University, Chiang Mai 50200, Thailand; wandee.th@cmu.ac.th; 5 Center of Excellence in Materials Science and Technology, Chiang Mai University, Chiang Mai 50200, Thailand; 6 Department of Mechanical Engineering, Faculty of Engineering, Chiang Mai University, Chiang Mai 50200, Thailand; tanongkiat_k@yahoo.com; 7 Center of Excellence in Microbial Diversity and Sustainable Utilization, Chiang Mai University, Chiang Mai 50200, Thailand; 8 Academy of Science, The Royal Society of Thailand, Bangkok 10300, Thailand

**Keywords:** bio-based building material, mycelium technology, standard testing guidelines, sustainability, Sustainable Development Goals (SDGs) 8 and 9

## Abstract

Mycelium-bound composites (MBCs) represent a promising advancement in bio-based building materials, offering sustainable alternatives for engineering and construction applications. This review provides a comprehensive overview of the current research landscape, production methodologies, and standardization ideas related to MBCs. A basic search on Scopus revealed over 250 publications on MBCs between 2020 and 2024, with more than 30% focusing on engineering and materials science. Key studies have investigated the physical and mechanical properties of MBCs, optimizing parameters such as substrate type, fungal species, incubation time, and post-processing to enhance material performance. Standardizing the inspection of MBC properties is crucial for ensuring quality and reliability. Various testing standards, including those from the American Society for Testing and Materials (ASTM), the International Organization for Standardization (ISO), the Japanese Industrial Standard (JIS), European Standards (EN), Deutsches Institut für Normung (DIN), and the Thai Industrial Standards Institute (TIS), are utilized to evaluate density, water absorption, compression strength, tensile strength, insulation, and other critical properties. This review highlights the distinction between lab-scale and apply-scale testing methodologies, emphasizing the need for comprehensive evaluation protocols. Additionally, the production process of MBCs involves critical steps like substrate preparation, fungal species selection, and mycelium growth, necessitating the implementation of good manufacturing practices (GMPs) to ensure consistency and quality. The internal and external structures of MBCs significantly influence their performance, necessitating standardized inspection methods using advanced techniques such as scanning electron microscopy (SEM), X-ray computed tomography (CT) scanning, and surface profilometry. By establishing robust inspection protocols and production standards, the industry can enhance the reliability and adoption of MBCs, contributing to innovations in materials science and promoting environmental sustainability. This review underscores the importance of interdisciplinary collaboration, advanced characterization tools, and regulatory frameworks to address challenges and advance the field of MBCs.

## 1. Introduction

MBCs are emerging as an innovative material in the field of engineering due to their unique properties and sustainable production methods [1]. MBCs, derived from the root-like structures of fungal mycelium combined with various lignocellulosic substrates, present an eco-friendly alternative to traditional synthetic materials, offering benefits such as biodegradability, low energy consumption during production, versatile properties, and the ability to be sourced from agricultural waste [2,3,4]. These composites exhibit unique characteristics and demonstrate advantageous physical and mechanical properties, such as being cost-effective, safe, biodegradable, and environmentally friendly, especially when compared to traditional building and construction-related materials. This makes them suitable for a wide range of applications, particularly as bio-based building materials, including construction materials, household items, interior materials, semi-structural elements, along with furniture [5,6,7].

Despite the promising potential of MBCs, the lack of standardized inspection protocols and production methods poses significant challenges and limitations to their widespread adoption and commercialization [7,8]. The diverse nature of mycelium, coupled with variations in growth substrates and environmental conditions, results in composites with inconsistent properties [3]. This variability can impact the reliability and performance of MBCs, hindering their acceptance in critical applications where material consistency is paramount [8].

To address these challenges, it is imperative to develop comprehensive guidelines for the inspection of MBC properties and the standardization of production methods. Such guidelines would not only ensure the quality and uniformity of MBCs but also facilitate regulatory approval and marketing expansion. This review paper aims to provide a thorough examination of the current state of research on MBCs, highlighting the key factors influencing their properties and performance. It will also explore existing inspection techniques and production practices, identifying gaps and proposing strategies for standardization.

By incorporating the latest findings and offering a roadmap for future research, this review seeks to advance the understanding and application of MBCs in engineering. The establishment of standardized inspection and production protocols will be crucial in unlocking the full potential of MBCs, paving the way for their integration into a sustainable and innovative materials landscape.

## 2. Collection of Information

**Figure 1:**
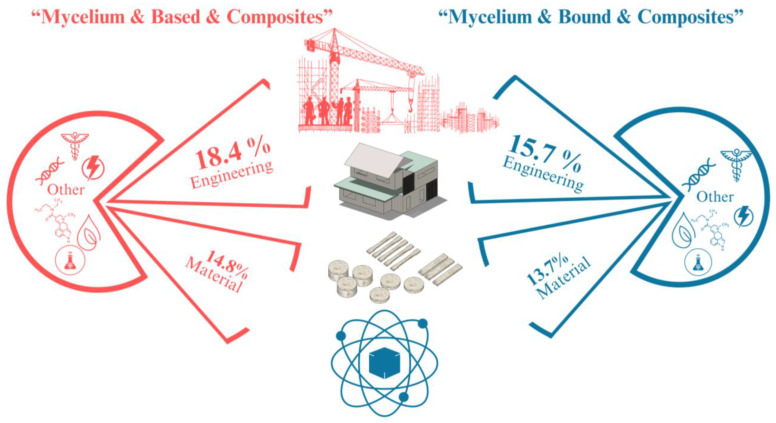
Subject-specific documents related to the engineering and materials science fields of MBC manufacturing. Created by Google SketchUp program version 8 for Windows.

A basic search was conducted on Scopus using the keywords “mycelium & based & composites” and “mycelium & bound & composites” between 2020 and 2024 (https://www.scopus.com/search/form.uri?display=basic#basic; accessed on 27 August 2024). These keywords provide a comprehensive definition of MBC research, helping to gather key findings and identify important points related to testing standards for various properties. The search showed more than 250 publications on MBCs. Over 30% of these publications are in the fields of engineering and materials science (Figure 1), emphasizing the study of various material properties and production processes. For example, several studies have investigated the physical and mechanical properties of MBCs to identify the most suitable fungal species and substrate types for production [9,10,11,12,13]. One such study by Mbabali et al. [14] investigated using *Pleurotus ostreatus* as a binder for rice husks and sawdust to produce foam-like material with potential applications in insulation, packaging, and construction. The study utilized the Box–Behnken experimental response surface methodology to optimize the effects of substrate type, water content, and incubation time on the material’s physical, mechanical, thermal, and fire safety properties. The results showed that rice husk composites exhibit superior deformation resistance, fire-retardant properties, and reduced water absorption compared to sawdust composites, making them ideal for thermal insulation and lightweight packaging. The study concluded that MBCs produced from these substrates are sustainable and hold strong commercial potential. Similarly, Kohphaisansombat et al. [15] aimed to improve the properties of MBCs derived from *P. ostreatus* using spent coffee grounds as the main substrate, combined with natural pineapple fiber. This study focused on physical and mechanical properties such as flexural strength, water absorption, swelling, and sound absorption, along with fire resistance tests confirming the non-flammable properties of the MBCs. Aiduang et al. [12] examined the properties of MBC materials, focusing on mechanical properties (compressive, flexural, impact, and tensile strength) and physical properties (density, shrinkage, water absorption, and thermogravimetric analysis). This study utilized four different fungal species (*Ganoderma fornicatum*, *Ganoderma williamsianum*, *Lentinus sajor-caju*, and *Schizophyllum commune*) combined with three types of lignocellulosic residues (sawdust, corn husks, and rice straw). The findings indicated that variations in both the type of lignocellulosic residues and fungal species could significantly influence the properties of the obtained MBCs, leading to composites with diverse and beneficial characteristics. Furthermore, many other studies have researched MBC production and investigated their properties for potential use as alternative materials in building and engineering applications [11,15,16,17,18,19].

In recent years, numerous MBCs have been developed as materials for engineering models. For example, Früchtl et al. [20] explored the potential of MBCs as a sustainable alternative to wood or rigid foam in pultruded glass fiber-reinforced plastic (GFRP) sandwich profiles. This study also evaluated the performance and environmental sustainability of this composite material through mechanical testing and life cycle assessment (LCA). Additionally, Abdelhady et al. [21] aimed to expand the design potential of MBCs to create advanced systems for a new material culture in architecture. They proposed a design method for fabricating small-scale MBC components for use in modular systems, enabling the construction of lightweight structures without additional reinforcement. This construction system reduces the weight of the structure and uses fewer materials compared to traditional construction methods, thereby lowering the carbon footprint and resulting in a reduced environmental impact on the construction industry [22].

Overall, the potential directions for MBC materials in engineering applications are very promising, particularly in supporting sustainable solutions and developing green building and construction materials [3]. Although, MBCs have been recognized as possible alternatives to traditional building construction materials due to their low cost, safety properties, and environmental benefits [2,23]. However, a review of processing techniques has shown that MBCs exhibit limitations, such as low strength and high water absorption, which affect their reliability and practical applicability [2,24]. Elsacker et al. [25] suggested that these issues could be addressed through careful consideration of key factors (including fungal species/strains and substrate types) and by optimizing growth conditions. Similarly, previous studies have synthesized insights into various strategies for strengthening and enhancing MBCs, particularly through mechanical compaction and strategic material placement. Mechanical compaction increases the density and structural integrity of MBCs, improving their load-bearing capacity [4,26]. Additionally, placing the material strategically in structures can optimize the strength-to-weight ratio, making MBCs suitable for larger and more ambitious architectural applications despite their natural limitations in compressive strength [4]. Addressing these challenges is a key focus of progressing research [2]. Therefore, creating and establishing standards for both production and inspection is extremely important for improving the practical applicability of MBCs. To aid in understanding, this review includes a comprehensive list of relevant symbols and abbreviations, summarized in Table 1.

## 3. Standardization

### 3.1. Inspection of Properties

Material inspection involves examining and assessing various properties of materials to ensure they meet specific standards, specifications, or quality requirements. These inspections are commonly used for quality assurance (QA) in various industries [27]. Table 2 presents a comprehensive overview of the various test standards used to evaluate the properties of MBC materials. The application scale verifies material properties by clearly specifying the goals of its application, emphasizing comparisons between standard wood panels, particleboards, hard cellular plastics, and other construction-related materials. The lab and pilot scales check basic properties such as density, moisture content, water absorption, thickness swelling, compressive strength, tensile strength, impact resistance, modulus of elasticity, flexural strength/bending strength, internal cohesive force, shear force, thermal insulation, sound absorption, sound insulation, environmental impact assessment, flammability, termite resistance, and biodegradability. Determining the suitability of an MBC for future applications is of great importance. Each property is associated with multiple testing standards related to various materials commonly used in the building and construction sectors, such as wood-based materials, plastics, boards, fibers, cements, metallic materials, acoustical materials, as well as core materials. These testing standards reflect the diverse methodologies employed in research efforts to evaluate MBCs.

Numerous standard methods have been used to evaluate MBC properties, including those from the ISO, JIS, TIS, EN, and DIN. However, the most frequently used standards are from the ASTM. For instance, density assessment uses ASTM D1622, ISO 9427, and JIS A 5908 standards [12,14,29], demonstrating global standardization. Similarly, water absorption testing encompasses ASTM, JIS, and ISO standards [12,14,15,29,30,31,32,33,34,35], showcasing a blend of international and industry-specific guidelines. Compression strength is evaluated using standard methods such as ASTM C109, ASTM D1621, ASTM D2166, ASTM D3501, DIN 50134, EN 1015, and ISO 844 [14,15,30,37,38,39,40]. This diversity underscores the importance of a multifaceted approach in evaluating composite properties, accommodating different testing environments, and regional preferences.

Upon closer examination, Table 2 reveals a clear distinction between lab-scale, pilot-scale, and apply-scale testing methodologies. Lab-scale and pilot-scale tests are typically more controlled and detailed, used for the precise measurement and analysis of material properties, such as ASTM D570 for the water absorption of plastics, ISO 844 for the compression properties of rigid cellular plastics, and ISO 16929 for determining the degree of disintegration of plastic materials under defined composting conditions. In contrast, apply-scale tests are designed for practical, real-world applications and performance evaluations, such as JIS A 5908 for particleboards and ISO 15148 for the hygrothermal performance of building materials. This distinction ensures that materials are evaluated both in controlled environments and under conditions that mimic actual usage scenarios, providing a comprehensive understanding of their performance and suitability for various applications. The comprehensiveness and diversity of these standards underscore the importance of multi-faceted testing in ensuring material reliability and safety in construction and manufacturing. By offering a robust framework for selecting appropriate standards, the table supports the advancement of MBCs, promoting innovation and reliability in this emerging field.

Referencing studies that utilize these standards enhances the credibility of the research and highlights the practical application of each standard. Researchers and practitioners in the field of MBCs can use this table as a guideline and valuable resource to select appropriate testing standards based on their specific objectives and the properties they aim to evaluate. Furthermore, this table can serve as a foundation for standardizing testing protocols within the industry, fostering consistency and comparability across different studies and laboratories. Ultimately, it facilitates informed decision-making and advances the understanding of MBCs’ performance characteristics.

### 3.2. Production Methods

**Figure 2:**
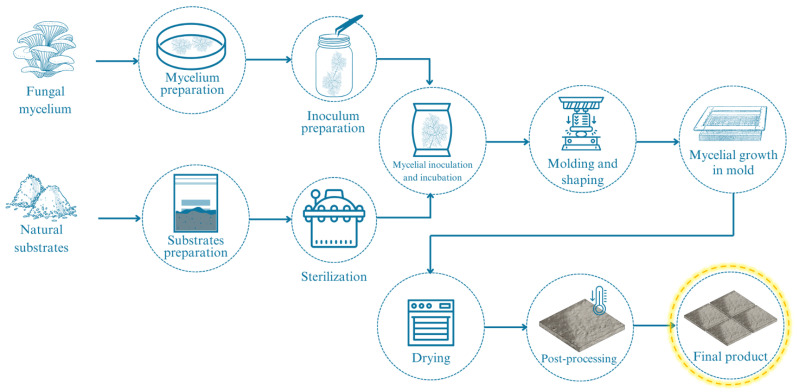
An overall biotechnological process is generally used to produce MBCs (modified from Elsacker et al. [25] and Almpani-Lekka et al. [60]). Created by Google SketchUp program version 8 for Windows.

The production process of MBCs involves several key steps (Figure 2), including substrate preparation, fungal species selection, sterilization, inoculation, incubation, molding and shaping, mycelial growth in mold, drying, and post-processing. This systematic process utilizes biotechnological approaches that leverage the natural growth of fungal mycelium [1,58]. Each step, from substrate preparation to drying and maintenance, is crucial to ensure the quality and performance of the final MBC products [59]. By understanding and optimizing these processes, MBCs can be effectively utilized as eco-friendly alternatives in various applications, promoting the principles of the bio-circular-green economy (BCG) [8,29].

However, standardization in the production processes is essential to establish good manufacturing practices [61], especially if MBC production is scaled up. Ensuring consistency and quality control (QC) at each stage will be vital for the successful large-scale production of these sustainable materials. Creating standards for the production of MBCs requires meticulous attention to various aspects to ensure quality, safety, and efficiency [62].

**Figure 3:**
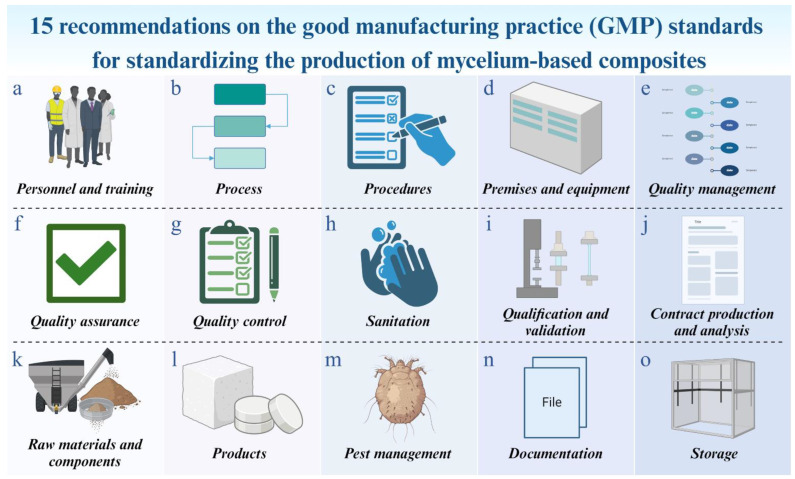
Recommendation on good manufacturing procedure (GMP) guidelines to standardize MBC production for applications involving bio-based building materials. Created by BioRender.com (https://www.biorender.com/; access date: 5 June 2024 [68]).

One compelling approach involves adapting and implementing guidelines from the good manufacturing practice (GMP) standard (Figure 3). These guidelines can encompass key areas such as personnel, training, processes, procedures, premises, equipment, quality management, assurance, and control, as well as sanitation, hygiene, qualification, validation, product recalls, contract production, analysis, quality audits, supplier audits/approval, raw materials, products, pest management, storage, and documentation [63,64,65,66,67]. Strict adherence to these guidelines, along with the establishment of standardized protocols, can significantly enhance the production standards of MBC materials in the future, facilitating more efficient manufacturing processes.

- Personnel and training (a): People are the most crucial factor in ensuring product quality in the manufacturing process [63]. All personnel involved in production should receive comprehensive training on essential parameters [64,65]. This includes handling mycelium, substrate materials, equipment, and following established procedures that constitute the main manufacturing process for MBCs [10,23,69]. Understanding the mycelium networking process is crucial, as it provides insights into how these composites achieve their unique properties. Knowledge of the substrate composition and its role as a supportive structure is also important, as it affects the adhesive properties of the mycelium [4]. Additionally, optimizing growth conditions through carefully regulating temperature, humidity, aeration, gas exchange, incubation time, light exposure, and nutrient concentrations, can enhance the adhesive qualities of mycelium [23,59]. To improve understanding of the situations that result in optimum strong characteristics, techniques for training, simulating, and machine learning can be applied [4,70]. Responsibilities should be clearly defined, understood, and documented as written job descriptions, with each person assigned tasks based on their capability, knowledge, and experience [63,71]. Additionally, strict adherence to personal hygiene practices, such as handwashing and wearing clean clothing, is essential to prevent contamination throughout the manufacturing process, which should always maintain sterile conditions [63,72]. Importantly, assessing their performance is also essential to raising skill, productivity, and efficiency [65].
- Process (b): In general, using MBCs in building materials and their integration into architecture involves a classification method based on the species of mycelium, substrate combination, supporting structure, and post-treatment processes [4,73]. To ensure consistent production, critical steps in the production process must be identified, and control procedures should be flexible enough to be adjusted as necessary [74]. The production process should be well-defined and documented, detailing each step from the initial process to the final product [65]. For MBC production, this typically includes steps from substrate preparation to final product storage. Comprehensive and standardized processes should be defined for the production of MBCs, including substrate preparation, inoculation, mycelium cultivation, molding, post-processing, until storage [25,59]. Monitoring and controlling process parameters, such as temperature, humidity, aeration and gas exchange, incubation time, and light exposure, are essential to ensure consistency and reproducibility [1,59]. These parameters should be well-documented and regularly checked by personnel. Regular evaluations should be conducted to ensure compliance with practices and organizational requirements, reducing the risk of contamination and ensuring product safety [64]. Additionally, the documentation of all activities and the reporting of deviations when they occur is crucial. This comprehensive approach helps in the early detection of errors and other deviations, reducing potential losses for the manufacturer [64,75,76].
- Procedures (c): A procedure is a set of guidelines designed to achieve consistent results in a critical process or part of a process. Employees in each manufacturing process must adhere to these rules and procedures to ensure smooth operations. These guidelines should be communicated to all employees and followed consistently [65,77]. All procedures must be created and documented to provide clear instructions on what each process must do and how it must meet the required standards. These procedures must be explicitly indicated and consistently followed [78]. Instructions and procedures should be written in clear and unambiguous language, specifically tailored to the facilities provided. Any deviation from standard procedures should be reported immediately and investigated [63,65]. This principle is crucial because if a defect or potentially unsafe output occurs in a lot, there is no need to inspect the entire production. Proper documentation allows staff to quickly identify the source of the problem and address it promptly [79]. For each step in the production process of MBCs, from substrate preparation, inoculation, and growth monitoring to molding and dehydrated processing [25,59,60], procedures should be regularly reviewed and updated to reflect new insights or technological advancements. These procedures are necessary to ensure the composite’s structural integrity and prevent any biological activity that could affect its qualities. Based on their purpose, MBCs might need additional processing, like machining or pressing to obtain the appropriate density or coatings to improve durability or visual appeal. Additionally, optimizing substrate composition and production methodology is important for maximizing the efficiency of MBCs [4].
- Premises and equipment (d): Generally, any building or structure, including any machinery, apparatus, engineering systems, or other objects physically affixed and integrated into it, is referred to as the premises. Machines and other devices used for assistance, prevention, treatment, or measurement are referred to as equipment [64]. Primally, premises must be located, constructed, adapted, designed, and maintained to suit the operations to be carried out. The layout and design of premises should aim to minimize the risk of errors and allow for effective cleaning and maintenance to prevent cross-contamination, and the buildup of dust or dirt, and to avoid any adverse effect on the quality of final products. The layout, ventilation, and water supply must always be in favorable condition [63,64,79,80,81]. Additionally, maintenance and storage areas should be separated from the production area, and storage areas must have sufficient capacity to store products in a well-organized and orderly manner [64]. Meanwhile, manufacturing equipment should be capable of producing materials or products that meet the required quality standards. Equipment must be designed and built to be thoroughly cleaned and sterilized, as well as used efficiently. Surfaces that come into contact with samples should have polished finishes and be smooth to minimize contamination, ease cleaning, and facilitate use. Equipment must withstand repeated, thorough cleaning. All manufacturing equipment must be thoroughly cleaned or sterilized between batches [63,82]. Moreover, all equipment should be properly placed or stored and regularly checked to ensure it is fit for producing consistent results and to prevent various risks [65]. Importantly, all facilities and equipment must have properly documented cleaning processes. Measures to prevent cross-contamination must be in place, along with written instructions for inspections [64,80]. Routine inspections of equipment and machinery, as well as sanitation inspections, must be carried out [83]. With accessible supplies, cleaning and maintenance are easier. Staff should also have adequate facilities and tools to maintain personal hygiene [79]. For the production of MBCs, the manufacturing facility must be designed to support the optimal growth conditions for mycelium and to ensure contamination control. The premises should be clean, and growth conditions must be carefully controlled to enhance the binding qualities of mycelium [4,23]. Equipment used in all stages of production, such as autoclaves, molds, unidirectional press machines, incubators, along with drying machines, must be maintained in good working condition to prevent contamination and ensure reproducibility.
- Quality management (e): The key to creating a successful manufacturing process is first to understand all the basic concepts [79]. Quality management ensures that all operations adhere to GMP guidelines, a fundamental concept accepted across various industries [82]. Implementing a quality management system (QMS) to oversee all aspects of production, ensuring compliance with standards and continuous improvement, is mandatory for many industries, especially those seeking GMP certification [79]. Similarly, ISO 9001:2015 is another essential standard that provides comprehensive requirements related to QMS. Compliance with ISO standards, and regularly setting and reviewing quality objectives to align with production goals and regulatory requirements, demonstrates an industry’s ability to consistently produce high-quality products in line with regulatory requirements, including GMPs [79,84]. In MBC manufacturing, a strong QMS should be implemented to manage the entire production process. This includes QA and control measures to monitor the consistency and performance of the obtained MBCs. Regular quality checks and audits should be conducted to ensure compliance with internal standards as well as any external regulations that may emerge in the future.
- Quality assurance (f): QA is a broad concept encompassing all factors that influence product quality, ensuring that final products meet the required standards. It involves organized arrangements aimed at ensuring products are of the necessary quality for their intended use, incorporating principles such as GMP along with factors like product design and development [63]. In the production of MBCs, QA might face challenges due to batch variability arising from different fungal growth patterns [85]. Moreover, QA still involves systematic activities within a QMS to fulfill product quality requirements [86]. Guidelines adapted from GMP standards can provide a comprehensive framework for QA in MBC production. This includes overseeing production and control operations, implementing necessary controls on starting materials, ensuring correct processing, and checking of the finished product, establishing arrangements for reporting, investigating, and recording deviations, along with conducting regular evaluations of product quality [63]. Implementing these comprehensive QA guidelines, derived from GMP standards, might help ensure consistent production of high-quality MBCs for various applications, particularly in engineering fields where dimensional stability is essential.
- Quality control (g): Producing high-quality MBCs presents challenges due to the variations in fungal species and substrate types used [1,8,36]. Both the choice of fungal species and substrate type, along with manufacturing methods, significantly affect the quality of MBCs. However, the fungal species typically have a greater impact on the final composite properties than the substrate type [1,33]. Therefore, developing appropriate QC measures is essential to address these challenges and ensure consistent product quality [87]. Standardization is crucial for maintaining consistent material properties across different batches and meeting industry standards [88]. Some companies are working to establish standardized methods and QC measures for mycelium production [89]. Adapting guidelines from GMP standards might provide a structured approach for QC in MBC production. Generally, QC involves sampling, specifications, testing, and documentation to ensure that all necessary tests are carried out. It is not limited to laboratory operations but influences many decisions concerning product quality [63]. Additionally, conducting systematic QC activities involves carrying out protocols for maintaining, or storing materials [90]. By adhering to QC guidelines derived from GMP standards, producers may consistently deliver high-quality MBCs that meet the stringent requirements of applications.
- Sanitation (h): Adopting GMP standards might offer a comprehensive framework for sanitation in MBC production. Primarily, the scope of sanitation covers personnel, premises, equipment and apparatuses, production materials and containers, cleaning and disinfection products, and anything that could become a source of contamination to the product. Potential contamination sources should be eliminated through an integrated and comprehensive program of sanitation and hygiene [63,91]. To eliminate potential sources of contamination, an integrated and comprehensive sanitation and hygiene program should be implemented. Areas, surfaces, and equipment involved in MBC manufacturing must be kept clean, as dirt and the microbes it harbors must not come into contact with the products. Several research works on MBC production and other related fields have indicated that these surfaces can be cleaned by applying a cleaning agent, such as 70–75% ethanol, sodium hypochlorite, or hydrogen peroxide, followed by rinsing with autoclaved water [29,59,63,92]. Additionally, UV-C sterilization, which employs light to sterilize surfaces and air by disrupting the Deoxyribonucleic acid (DNA) of microorganisms, serves as another effective option for sterilizing some tools, molds, working surfaces, and air within clean rooms [59,93].
- Qualification and validation (i): The guidelines for qualifying and validating MBCs in engineering offer a systematic approach to ensure production reliability and consistency. Adapting these practices from GMP standards may help establish robust protocols specific to MBC production. Qualification and validation are essential for material QA and processes to meet regulatory requirements and produce consistent products. Generally, the qualification process includes design qualification (DQ), installation qualification (IQ), operational qualification (OQ), and performance qualification (PQ), each requiring detailed documentation and verification [63]. Validation confirms that the production consistently meets predetermined specifications. Documentation, including protocols and test results, is crucial for tracking various activities. Continuous monitoring and periodic revalidation are necessary to maintain compliance and adapt to any changes in regulations or technology [94]. Adhering to these guidelines can achieve high-quality, reliable MBC production, meeting regulatory requirements and benefiting engineering advancements. In this context, all aspects of production, including processes, equipment, materials, and fungal species or strains, should undergo rigorous qualification and validation. This ensures that growth conditions for mycelium, equipment performance, and the consistency of the final composites meet the required standards. Importantly, the validation activities should be documented and reviewed periodically.
- Contract production and analysis (j): MBCs in engineering should be contract manufactured and analyzed under strict quality, safety, and compliance criteria. These comprehensive guidelines might be developed for contract manufacturers and analytical labs by adapting based on GMP standards. Contracts should clearly define roles, responsibilities, and expectations, ensuring facilities and expertise align with requirements. It should be ensured that contract manufacturers have the necessary facilities, equipment, and expertise to meet specified requirements. Protocols for material handling, processing, and storage to prevent contamination and maintain product integrity should be established. Regular audits and inspections of manufacturing facilities verify compliance with quality standards [82]. Analytical methods and specifications for testing [95], covering the physical, mechanical, chemical, and microbiological attributes that are essential for assessing MBC products, should be defined [6,10,12,45,59]. Analytical labs have validated methods and qualified personnel for accurate and reliable analyses. Consistent protocols for sample collection, preparation, and testing should be established. Analytical methods for accuracy, precision, and specificity should be validated before practice use [63,82,95]. By following these guidelines, stakeholders can ensure consistency, quality, and safety in MBC production, supporting the advancement of MBCs in engineering applications.
- Raw materials and components (k): The availability of raw materials and the simplicity of producing materials based on microbes can minimize costs and ensure the reliable manufacturing of high-quality products [8]. Guidelines adapted from GMP standards encompass the selection, handling, storage, and QC of these materials [82]. Raw materials, including fungal mycelium (strains) and substrates like agricultural or forestry wastes, must be sourced from reliable suppliers with documented quality practices. Whenever possible, these materials should be sourced close to the production site to avoid transportation issues [73,96]. Additionally, selecting substrates rich in cellulose, hemicellulose, and lignin, known to enhance adhesion, can improve the binding efficiency of mycelium and the mechanical properties of the obtained MBCs [4,10,23]. Detailed specifications, based on scientific data, should be established for moisture content, particle size, and absence of contaminants. Materials should be stored in dry, temperature-controlled environments to prevent degradation and contamination. Rigorous inspection and assessment, including visual and laboratory testing, are necessary upon receipt to confirm compliance with specifications. Non-conforming materials should be stored separately to prevent accidental use and contamination [82]. By adhering to these guidelines, manufacturers can ensure consistent production of high-quality MBCs, supporting the development of reliable materials for engineering applications.
- Products (l): Developing standards to produce MBCs for engineering applications and adherence to guidelines derived from GMP standards may be crucial. These guidelines ensure the quality and efficacy of the final products [64]. A key challenge is ensuring that biomaterials meet performance standards and industry requirements [97]. Consistent performance, durability, and safety are essential for gaining the trust and confidence of construction professionals and building developers. Creating comprehensive attributes and specifications for MBC materials involves defining parameters such as dimensions, characteristics, strength, safety, and environmental sustainability. Robust QC testing protocols are essential for evaluating the quality and consistency of MBC products. Tests should cover physical, mechanical, chemical, and biological properties, as well as dimensional stability [98]. Stability testing to assess the shelf-life and storage conditions of products is also important, evaluating factors such as temperature, humidity, and light exposure [99]. By adhering to these guidelines adapted from GMP standards, manufacturers can ensure the consistent production of high-quality MBC products that meet regulatory requirements and the needs of engineering applications.
- Pest management (m): Effective pest management is essential, following GMP standards [66]. To maintain the quality of MBCs in engineering applications, these guidelines focus on preventing pest infestations that could compromise the integrity of MBCs. Key aspects include implementing measures to prevent pests from entering the production area, such as sealing entry points and using appropriate pest control methods or pest-resistant materials approved for use in production areas. Regular inspections and monitoring are essential to detect and address pest issues promptly. Non-chemical control methods, such as traps and barriers, should be prioritized, with chemical control methods used as a last resort and in compliance with regulations. All pest management activities should be documented, including records of inspections, treatments, and any corrective actions taken. Additionally, training employees on pest management practices and maintaining cleanliness in production areas are vital components of an effective pest management program for production processes [66,100]. Nonetheless, prior research suggests that guayule resin and various kinds of natural oils can be effective treatments for enhancing pest resistance in MBCs, particularly for improving resistance to termites [55]. Implementing these methods can further improve pest resistance and support the overall integrity of the production process.
- Documentation (n): The main objective of the documentation system is to establish, control, monitor, and record all activities that directly or indirectly impact the quality of products [63]. Comprehensive recording is crucial for QC, especially when developing products for engineering purposes. Guidelines adapted from GMP standards emphasize the importance of comprehensive documentation throughout the production process. This includes detailed records of raw material sourcing, production procedures, QC measures, and storage conditions. All documentation must be accurate, up-to-date, and easily accessible for review [64,101]. Any deviations from standard procedures or quality specifications must be documented, along with corrective and preventive actions taken. Clear and thorough documentation ensures traceability, facilitates QA [63], and supports regulatory compliance in MBC production for future engineering applications. In this case, detailed records should be maintained for each stage of production. This includes documenting substrate and ingredient sources, growth conditions, molding and drying requirements, the post-processing process, testing results, and suitable storage. Proper documentation not only facilitates traceability and accountability but also supports continuous improvement in the production process.
- Storage (o): To ensure the quality of MBCs in engineering applications, stringent storage guidelines are crucial, adapted from GMP standards. These guidelines focus on storing finished MBCs under controlled conditions to prevent degradation and contamination. Maintain a controlled environment with monitored temperature and humidity levels (ideally below 60%, preferably between 30–50%, and at a specified temperature range) to prevent microbial growth [59]. Adequate ventilation is essential to prevent moisture buildup. Implement pest control measures to prevent material damage. Detailed storage records should be maintained, including conditions, monitoring data, and deviations from standard conditions [82]. These guidelines ensure the integrity of composites during storage, contributing to the reliability and effectiveness of the final products.

## 4. Inspection of Composite Structures

The development of biocomposites as sustainable materials for engineering applications necessitates rigorous inspection protocols to ensure their quality and performance [98]. The internal and external structures of MBCs significantly influence their physical and mechanical properties, durability, and suitability for various applications [15,44]. Hence, standardizing the inspection methods for these structures is crucial for advancing the use of MBCs in industry.

The internal structure of MBCs is primarily characterized by the mycelial network, porosity, and the distribution of the substrate material [59]. In materials science and engineering, these characteristics are directly linked to important properties, such as composite strength and material density [23,28,94,102,103]. To standardize the inspection of the internal structure, the following guidelines are proposed. Firstly, SEM and transmission electron microscopy (TEM) can be used to examine the mycelial network at micro and nano scales. These techniques provide detailed images of the internal morphology, revealing the density and connectivity of the mycelium [5,54]. Secondly, porosity analysis can be conducted using techniques such as mercury intrusion porosimetry (MIP) and water permeability to quantify pore size distribution and porosity levels. This analysis helps in understanding the composite’s insulation properties and potential for fluid absorption [11,104]. Thirdly, CT scanning can be implemented to non-destructively visualize the internal structure in three dimensions. This technique allows for the assessment of the uniformity of the mycelial growth and the distribution of substrate materials [11,105]. For a simpler inspection, a basic microscope can be used to examine a small sample of the composite, focusing on key aspects such as the density and connectivity of the mycelial network, as well as the presence of mycelium. This helps confirm that the MBCs produced by the mycelium are effectively acting as a binding agent, while also ensuring even distribution of the substrate within the matrix [4].

The external structure of MBCs encompasses surface texture, homogeneity, aesthetics, and integrity [45,106]. Establishing standardized protocols for inspecting the external structure involves several key guidelines. Firstly, surface texture analysis typically utilizes techniques like SEM and surface profilometry to assess smoothness, porosity, and overall surface morphology [12,107]. Secondly, dimensional accuracy is ensured using precise measurement tools such as calipers or laser scanning to verify that the material adheres to specified dimensions and tolerances, essential for applications necessitating precise fits and finishes [108]. Additionally, visual and optical inspection methods, employing high-resolution cameras or optical microscopes, are employed to identify surface defects such as cracks, voids, uneven patterns, or irregularities that could compromise the composite’s performance [109]. Lastly, the external structure of MBCs is also related to color consistency [102]. Generally, the color and texture of mycelium can vary based on production factors, especially the fungal species used, substrate types, and production conditions, making it challenging to control the final appearance of composites [106,110]. Typically, a diverse array of appearances is available with bio-based materials, ranging from traditional and rustic options to more contemporary and modern designs. Considering potential biases from consumers against fungi and the specific characteristics of MBCs, questions about the material’s acceptance level among designers and future customers are fully justified [106]. Although some engineering limitations linked to color consistency might hinder standardization, the use of MBCs poses aesthetic challenges and opportunities for modern designs [24,108]. However, standardization efforts should focus on establishing protocols for composite production, including uniform substrate composition, growth conditions, and post-processing treatments, to minimize variability in color outcomes.

Developing standardized guidelines for the inspection of both the internal and external structures of MBCs is essential to ensure their reliability and performance in engineering applications [2]. These guidelines will provide a framework for consistent QC, facilitating the broader adoption of MBCs as sustainable materials. By establishing robust inspection protocols, the industry can better harness the potential of MBCs, contributing to innovations in materials science and promoting environmental sustainability.

## 5. Overview of Selected Inspection Standards

To select appropriate test standards for each property of MBC materials, several factors need consideration. Firstly, the intended application and regulatory requirements play a crucial role. For instance, if these materials are intended for construction purposes, standards such as ISO 22007-2 for thermal insulation property [44] and ASTM E1050 for sound insulation absorption should be prioritized [15,111]. These standards ensure that the materials meet specific performance criteria necessary for building safety and comfort. Similarly, environmental considerations, as highlighted in standards like DIN EN 15978 and DIN EN 15804 [50,51], are essential for assessing the sustainability and ecological impact of these materials, aligning with growing global concerns about environmental responsibility.

Secondly, the relevance of the testing standards to the specific properties of MBCs must be evaluated. For instance, properties like density, moisture content, and water absorption, critical for material stability and durability, are well-covered by standards such as ISO 9427, ISO 16979, and ASTM D1037, respectively [12,14,15,29,30,32,33]. These standards provide precise methodologies for accurate measurement, ensuring reliability and consistency in results. Similarly, mechanical properties like tensile strength, compression strength, and flexural strength, crucial for structural applications, can be assessed using a combination of ASTM and ISO standards [14,15,28,29,30,32,37,39,40,41,59], which are widely accepted in the industry for their rigor and comprehensiveness.

Overall, the selection of test standards for MBCs should be guided by a balance of regulatory compliance, intended application, and technical relevance. By prioritizing standards that address specific performance requirements, adhere to industry norms, and ensure environmental sustainability, researchers and practitioners can effectively evaluate and optimize these innovative materials for various applications, from construction to manufacturing and beyond.

## 6. MBC Applications for Bio-Based Building Materials

**Figure 4:**
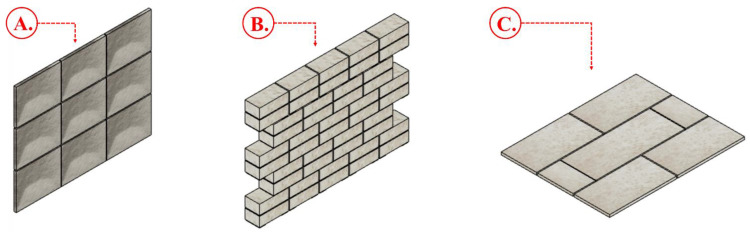
Composite models of MBCs for applications in bio-based building materials, such as boards (**A**), bricks (**B**), and floor tiles (**C**).

Standardized testing methods are essential in the manufacturing industry, playing a crucial role in ensuring material quality, durability, and safety. These methods involve examining material properties under various conditions to confirm compliance with established standards and specifications [112]. MBCs, known for their sustainable and biodegradable properties, require thorough characterization to establish their suitability for various applications, especially as building and construction materials in engineering fields [113,114]. These composites, such as boards, bricks, panels, and sheets (Figure 4), should be verified against building and construction material standards for each application. The listed standards, covering properties like density, water absorption, bending strength, compression strength, tensile strength, impact resistance, and thermal and sound insulation, offer a valuable reference point for assessing MBCs’ performance and durability.

By aligning mycelium material testing with these established standards, researchers and developers can ensure consistency and comparability with other conventional materials. For instance, using ISO 9427, ASTM D1622, and JIS A 5908 for density measurements [12,14,29] or ASTM D1037 for water absorption and the modulus of elasticity, can help benchmark MBCs properties against traditional wood-based panels and rigid cellular plastics [15,29,30,32,33]. This approach not only aids in optimizing the material’s properties through iterative testing and refinement but also facilitates regulatory compliance and market acceptance by demonstrating that MBCs meet or exceed existing industry standards.

## 7. Challenges and Future Perspectives

### 7.1. Challenges

- Biological variability: One of the primary challenges in standardizing MBCs is the inherent variability of biological materials [3]. Factors such as fungal species, substrate composition, and growth conditions can significantly influence the properties of MBCs [115], making it difficult to achieve uniformity across different collections and production sites.
- Complexity of material properties: MBCs possess complex properties, including mechanical strength, physical characteristics, and biodegradability, which are influenced by both the internal structure and external morphology [116]. Accurately characterizing and standardizing these multifaceted properties requires sophisticated analytical techniques and interdisciplinary expertise.
- Measurement techniques: The selection and implementation of appropriate measurement techniques for characterizing MBC properties can be challenging [7]. Ensuring that these techniques are accurate, reproducible, and applicable across various scales (from micro to macro) is essential for standardization efforts.
- Lack of established standards: Currently, there is a lack of established standards and reference materials for MBCs, which hampers the development of consistent inspection and production guidelines [2,7]. The absence of industry-wide benchmarks makes it difficult to compare results and ensure QC.
- Economic and technical constraints: Developing and implementing standardized inspection protocols and production methods can be resource-intensive, especially when extended production cycles are required. These constraints pose significant challenges for large-scale production [73]. Small-scale producers and new market entrants may also face economic and technical barriers in adopting these standards.
- Dynamic field of research: The field of MBCs is rapidly evolving, with continuous innovations in production methods and applications. Developing guidelines that remain relevant and adaptable to new advancements poses a significant challenge [116].
- Insect infestation: MBCs, being organic in nature, are susceptible to insect infestations and/or degradation by other pests [117], which can compromise their structural integrity and quality. Developing inspection guidelines to detect and mitigate insect-related issues is essential for maintaining the reliability of MBCs.
- Integration with international building codes: The challenge of integrating MBCs into existing international building codes lies in evaluating and validating them against standards primarily designed for traditional materials like synthetic foams and wood-based composites. Due to the unique properties of MBCs, careful consideration is needed, as they may not align with conventional testing methods.
- Scale-up and manufacturing consistency: Scaling up MBC production from the laboratory to an industrial scale while maintaining consistent quality and properties is a significant challenge. Variability in production processes can result in inconsistent composite performance, making it difficult to establish reliable inspection protocols.
- Environmental and health safety concerns: The biological nature of MBCs raises concerns about potential allergenic or toxic effects, as well as their environmental impact during production and disposal. Developing inspection guidelines that address these concerns requires extensive research and testing, including studies on their effects on plants, soil organisms, and water bodies.

### 7.2. Future Perspectives

- Interdisciplinary collaboration: Establishing standardized guidelines will benefit from collaboration across disciplines, including biotechnology science, materials science, architecture, engineering, environmental science, chemistry, and multidisciplinary [59]. Such collaborations can pool knowledge and resources to develop comprehensive and robust protocols. Moreover, enhanced cooperation between industries and regulatory bodies could also accelerate the creation of inspection guidelines by merging practical insights with cutting-edge research. These efforts might lead to the development of thorough, evidence-based standards that ensure the safe and effective use of MBCs in building applications.
- Advanced characterization tools and testing technologies: The adoption of advanced characterization tools and techniques, such as real-time growth monitoring and contamination tracking, can enhance the accuracy and reliability of property inspections for MBCs [118]. Additionally, various biotechnological tools, including industrial fermentation, strain improvement, recombinant DNA technology, gene editing, and gene silencing, have recently been experimentally applied in the design and development of MBCs [102]. These tools provide deeper insights into the microstructure and performance of MBCs.
- Development of reference materials: Creating and disseminating reference materials for MBCs will be crucial for standardization [102]. These reference materials can serve as benchmarks for QC and facilitate the comparison of results across different studies and production sites. As research progresses, there is potential to develop specific standards and certifications for MBCs, similar to those for other bio-based materials, particularly in building applications.
- Regulatory frameworks: Establishing clear regulatory frameworks and certification processes for MBCs will support the development and adoption of standardized guidelines. Regulatory bodies can play a key role in defining safety, performance, quality, and environmental standards for MBCs. Importantly, clear regulatory frameworks will provide confidence to manufacturers and consumers, promoting the commercialization of MBCs [119].
- Industry standards: The development of industry standards, led by organizations such as the ISO and ASTM, will provide a formalized approach to the standardization of MBC properties and production methods [6]. These standards can enhance the credibility and acceptance of MBCs in various applications, particularly in building materials, making it easier for companies to develop and market MBCs worldwide.
- Scalability and cost reduction: Research should focus on developing scalable production methods and cost-effective inspection techniques [103,120]. Making advanced analytical tools more affordable and accessible will facilitate the standardization process and broader adoption of MBCs.
- Sustainability and market adoption: Standardizing MBC production and inspection protocols will contribute to the sustainability and scalability of these materials. As standardized MBCs become more reliable and consistent, their market adoption is likely to increase, driving further innovation and investment in this field [59,62]. Future guidelines could incorporate sustainability indices, such as carbon footprint and LCA, into the inspection process for MBCs. This would not only ensure material performance but also align with the growing demand for sustainable building practices. Moreover, incorporating sustainability metrics into the guidelines can highlight the environmental benefits of MBCs. Standardized methods for assessing and reporting the ecological impact of MBC production and lifecycle will underscore their value as sustainable alternatives to conventional materials [121]. Developing guidelines that prioritize eco-friendly practices will align with global SDGs, advancing green building technologies, and overarching goals of engineering and sustainability [4,122]. However, these real-world applications will demonstrate the viability and advantages of MBCs.

## 8. Conclusions

This comprehensive review has focused on the need for standardized guidelines in the inspection and production of MBCs for bio-based building material applications. Through a detailed examination of over 200 publications on MBCs, we have identified key studies that focus on the physical and mechanical properties of these materials, demonstrating their potential in various engineering applications. These studies indicate the importance of material inspection and standardization. By employing a variety of testing standards, including those from the ISO, ASTM, JIS, and others, researchers have been able to rigorously assess properties such as density, water absorption, strengths, and thermal insulation. These standardized methods ensure consistency and comparability, which are essential for the broader adoption of MBCs in industry. Moreover, the production processes of MBCs, from substrate preparation to post-processing, require careful optimization to maintain quality and performance. Adapting GMP guidelines can significantly enhance the production standards of MBCs, ensuring that they meet industry requirements for quality, safety, and efficiency. Similarly, the development of inspection protocols for both internal and external structures of MBCs is critical. Techniques such as SEM, CT scanning, and porosity analysis provide detailed insights into the microstructure and performance of MBCs. Standardizing these inspection methods will facilitate QC and support the reliable use of MBCs in engineering applications. Selecting appropriate test standards based on intended applications and regulatory requirements is vital for the successful implementation of MBCs. By aligning testing protocols with established standards, researchers can ensure that MBCs meet specific performance criteria necessary for building safety and comfort. Despite the promising future of MBCs, several challenges remain, including biological variability, the complexity of material properties, along with economic and technical constraints. However, the field can advance significantly through interdisciplinary collaboration, the use of advanced characterization tools, and the development of regulatory frameworks and industry standards. Overall, the standardization of inspection and production methods for MBCs is essential for their successful integration into bio-based building material applications. By establishing robust guidelines and protocols, the industry can harness the full potential of MBCs, promoting sustainability and innovation in materials science and engineering.

## Figures and Tables

**Table 1:** A comprehensive list of notations utilized in this review.

Symbols and Letters	Description
MBCs	Mycelium-bound composites
%	Percentage
ASTM	American Society for Testing and Materials
ISO	International Organization for Standardization
JIS	Japanese Industrial Standard
EN	European Standards
DIN	Deutsches Institut für Normung
TIS	Thai Industrial Standards Institute
GMPs	Good manufacturing practices
SEM	Scanning electron microscopy
CT	X-ray computed tomography
SDGs	Sustainable Development Goals
&	And
GFRP	Glass fiber-reinforced plastic
LCA	Life cycle assessment
ID	Identification
BCG	Bio-circular-green economy
QMS	Quality management system
QA	Quality assurance
QC	Quality control
UV-C	Ultraviolet clean
DNA	Deoxyribonucleic acid
DQ	Design qualification
IQ	Installation qualification
OQ	Operational qualification
PQ	Performance qualification
TEM	Transmission electron microscopy
MIP	Mercury intrusion porosimetry

**Table 2:** The example standards for testing the properties of MBCs that have appeared in past studies.

Properties	Testing Standard ID	Standard Title (Description)	Level	Reference
Density	ISO 9427	Wood-based panels—determination of density	Lab-scale	[12,14,28,29]
	ASTM D1622	Standard test method for apparent density of rigid cellular plastics	Lab-scale	[30]
	JIS A 5908	Japan Industrial Standard: particleboards	Application-scale	[31]
Moisture content	ISO 16979	Determination of moisture content	Lab-scale	[14]
Water absorption	ASTM D1037	Standard test methods for evaluating properties of wood-base fiber and particle panel materials	Application-scale	[6,15,29,30,32,33]
	ASTM C272/272M	Water absorption of core materials	Application-scale	[12,14]
	JIS A 5908	Japan Industrial Standard: particleboards	Application-scale	[31]
	ISO 15148	Hygrothermal performance of building materials and products—determination of water absorption coefficient by partial immersion	Application-scale	[34]
	ASTM D570	Standard test method for water absorption of plastics	Lab-scale	[35]
Thickness of swelling	ASTM D1037	Standard test methods for evaluating properties of wood-base fiber and particle panel materials	Lab-scale	[33]
	TIS 876	Thai Industrial Standard: flat-pressed particleboard	Application-scale	[15]
	JIS A 5908	Japan Industrial Standard: particleboards	Application-scale	[31]
Compression strength	EN 1015	Methods of test for mortar for masonry—Part 11: determination of flexural and compressive strength of hardened mortar	Application-scale	[36]
	ASTM D3501	Standard test methods for wood-based structural panels in compression	Application-scale	[14,37]
	DIN 50134	Testing of metallic materials—compression test of metallic cellular materials	Lab-scale	[38]
	ASTM D1621	Standard test method for compressive properties of rigid cellular plastics	Lab-scale	[30]
	ASTM C109	Standard test method for compressive strength of hydraulic cement mortars	Lab-scale	[15]
	ISO 844	Rigid cellular plastics—determination of compression properties	Lab-scale	[39]
	ASTM D2166	Standard test method for unconfined compressive strength of cohesive soil	Lab-scale	[40]
Tensile strength	ASTM D1037	Standard test methods for evaluating properties of wood-base fiber and particle panel materials	Application-scale	[32,41]
	ASTM D 638	Standard test method for tensile properties of plastics	Lab-scale	[12,28,29]
	DIN 53292	Testing of sandwiches; tensile test perpendicular to the faces	Application-scale	[20]
Impact strength	ASTM D256	Standard test methods for determining the Izod pendulum impact resistance of plastics	Lab-scale	[12,29]
	ASTM D7136	Standard test method for measuring the damage resistance of a fiber-reinforced polymer matrix composite to a drop-weight impact event	Lab-scale	[42]
Modulus of elasticity	ASTM D1037	Standard test methods for evaluating properties of wood-base fiber and particle panel materials	Application-scale	[37]
	ASTM D3504	Standard specification for maleic anhydride	Lab-scale	[14]
	ISO 16978	Wood-based panels—determination of modulus of elasticity in bending and of bending strength	Application-scale	[41]
Flexural/bending strength	JIS A5908	Japan Industrial Standard: particleboards	Application-scale	[31]
	ASTM D1037	Standard test methods for evaluating properties of wood-base fiber and particle panel materials	Application-scale	[36,40,43]
	ASTM C78	Standard test method for flexural strength of concrete (using simple beam with third-point loading)	Lab-scale	[15]
	ISO 16978	Wood-based panels—determination of modulus of elasticity in bending and of bending strength	Application-scale	[41,44]
	ASTM D790	Standard test methods for flexural properties of unreinforced and reinforced plastics and electrical insulating materials	Lab-scale	[12,28,29]
	DIN 52186	Testing of wood; bending test	Lab-scale	[33]
	DIN 53293	Testing of sandwiches; bending test	Application-scale	[20]
Internal bonding	JIS A 5908	Japan Industrial Standard: particleboards	Application-scale	[31]
	EN 319:1993	Particleboards and fiberboards. Determination of tensile strength perpendicular to the plane of the board.	Application-scale	[41,45]
	ASTM D1037	Standard test methods for evaluating properties of wood-base fiber and particle panel materials	Lab-scale	[46,47]
Shear strength	DIN-EN 12090	Thermal insulating products for building applications. Determination of shear behavior.	Application-scale	[17]
	DIN EN 205	Adhesives—Wood adhesives for non-structural applications—Determination of tensile shear strength of lap joints	Application-scale	[43]
Thermal Insulation property	ISO 22007-2	Plastics—Determination of thermal conductivity and thermal diffusivity	Lab-scale	[48]
	ASTM C1113	Standard test method for thermal conductivity of refractories by hot wire (platinum resistance thermometer technique)	Lab-scale	[14]
	ISO 12664	Thermal performance of building materials and products—Determination of thermal resistance by means of guarded hot plate and heat flow meter methods—Dry and moist products of medium and low thermal resistance	Application-scale	[34]
Sound insulation absorption	ASTM E1050	Standard test method for impedance and absorption of acoustical materials using a tube, two microphones and a digital frequency analysis system	Application-scale	[15]
	DIN 4109:1989	Sound insulation in buildings; construction examples and calculation methods	Application-scale	[49]
Environmental assessment	DIN EN 15978	Sustainability of construction works—Assessment of environmental performance of buildings—Calculation method	Application-scale	[49]
	DIN EN 15804	Sustainability of construction works—Environmental product declarations-Core rules for the product category of construction products	Application-scale	[50]
	ISO 14040/14044	Environmental management—Life cycle assessment—Requirements and guidelines	Application-scale	[20,51,52]
Flammability	ISO 5660-1	Reaction-to-fire tests—Heat release, smoke production and mass loss rate	Lab-scale	[15]
	ISO 5660-1	Reaction-to-fire tests—Heat release, smoke production and mass loss rate	Lab-scale	[53]
	ASTM D7309	Standard test method for determining flammability characteristics of plastics and other solid materials using microscale combustion calorimetry	Lab-scale	[54]
Termite resistance	ASTM D3345-08	Standard test method for laboratory evaluation of wood and other cellulosic materials for resistance to termites	Lab-scale	[55]
Biodegradability	EN 13432:2000	Requirements for packaging recoverable through composting and biodegradation. Test scheme and evaluation criteria for the final acceptance of packaging	Lab-scale	[56]
	ISO 16929:2021	Plastics—Determination of the degree of disintegration of plastic materials under defined composting conditions in a pilot-scale test	Pilot-scale	[56]
	ISO 20200:2015	Plastics—Determination of the degree of disintegration of plastic materials under simulated composting conditions in a laboratory-scale test	Lab-scale	[57]
	ISO 846/2000	Plastics—Evaluation of the action of microorganisms	Lab-scale	[28]

## Data Availability

Data are contained within the article.
